# Restraint of appetite and reduced regional brain volumes in anorexia nervosa: a voxel-based morphometric study

**DOI:** 10.1186/1471-244X-11-179

**Published:** 2011-11-17

**Authors:** Samantha J Brooks, Gareth J Barker, Owen G O'Daly, Michael Brammer, Steven CR Williams, Christian Benedict, Helgi B Schiöth, Janet Treasure, Iain C Campbell

**Affiliations:** 1 Section of Eating Disorders, Institute of Psychiatry, King's College London, London, SE5 8AF UK; 2 Centre for Neuroimaging Sciences, Institute of Psychiatry, King's College London, London, SE5 8AF, UK; 3 Uppsala University, Department of Neuroscience, 75124 Uppsala Sweden

## Abstract

**Background:**

Previous Magnetic Resonance Imaging (MRI) studies of people with anorexia nervosa (AN) have shown differences in brain structure. This study aimed to provide preliminary extensions of this data by examining how different levels of appetitive restraint impact on brain volume.

**Methods:**

Voxel based morphometry (VBM), corrected for total intracranial volume, age, BMI, years of education in 14 women with AN (8 RAN and 6 BPAN) and 21 women (HC) was performed. Correlations between brain volume and dietary restraint were done using Statistical Package for the Social Sciences (SPSS).

**Results:**

Increased right dorsolateral prefrontal cortex (DLPFC) and reduced right anterior insular cortex, bilateral parahippocampal gyrus, left fusiform gyrus, left cerebellum and right posterior cingulate volumes in AN compared to HC. RAN compared to BPAN had reduced left orbitofrontal cortex, right anterior insular cortex, bilateral parahippocampal gyrus and left cerebellum. Age negatively correlated with right DLPFC volume in HC but not in AN; dietary restraint and BMI predicted 57% of variance in right DLPFC volume in AN.

**Conclusions:**

In AN, brain volume differences were found in appetitive, somatosensory and top-down control brain regions. Differences in regional GMV may be linked to levels of appetitive restraint, but whether they are state or trait is unclear. Nevertheless, these discrete brain volume differences provide candidate brain regions for further structural and functional study in people with eating disorders.

## Background

Anorexia nervosa (AN) is defined by deliberate calorie restriction, by weight loss (to less than 85% of normal weight), and by a pathological fear of weight gain, and can be divided into restricting (RAN) and binge-purging (BPAN) subtypes (Diagnostic and Statistical Manual, Fourth Edition, DSMIV [1]. People with RAN engage in pathological dietary restriction whereas those with the BPAN subtype eat, but engage in compensatory behaviours (bingeing or purging or both) to avoid weight gain. The aetiology of RAN and BPAN are unclear although there is an extensive literature on unique and shared physiological, developmental and psychological risk factors [2]. In general, people with AN are reported to have reduced global gray matter volume (GMV) and larger cerebrospinal fluid (CSF) volumes [3-5] particularly those with RAN [6]: moreover, these differences are present in adolescents with AN [3] that is, when neurodevelopment is incomplete. On recovery, some of these deficits resolve [3,7-9], which suggests that they are state related. Regional differences in GMV have also been reported in AN, such as reduced volumes in the cerebellum for those with longer- and hypothalamus for shorter duration of illness [10] bilateral hippocampus [11] anterior cingulate cortex (ACC) [7] extrastriate body (ESB) [12] insular cortex [13,14] and in temporal and parietal regions [15]. Additionally, some of these brain volume reductions are present in the early stages of the illness [6], and perhaps worsen as the disorder progresses, as there is some evidence that reductions in these brain regions correlate with reductions in Body Mass Index, e.g. [10]. Furthermore, regional reductions, particularly in the precuneus, seem to persist even after long-term recovery [16]. These effects seem particularly prominent in females with the restricting type of AN [6]. There has only been one VBM study to date examining those with bulimia nervosa [17] and direct comparisons between AN and bulimia nervosa were not done. Direct comparisons of brain volume using VBM between those who binge (e.g. bulimia nervosa) and AN have only been done to date in those who have recovered from an eating disorder, and show normalization in brain volumes [9]. A recent region of interest study using Magnetic Resonance Spectroscopy (MRS) has shown that different brain metabolite correlations were found in the ACC in women with AN compared to bulimia nervosa, in that the latter showed correlations of metabolites with 'drive for thinness' [18]. However, no VBM study has directly compared brain volume differences between the subtypes of AN and examined the effect of differential levels of dietary restraint on brain volume.

The main aim of this study was to compare structural brain volumes in women with AN versus HC, and to examine whether differential levels of dietary restraint are related to global and regional gray matter reductions. Furthermore, as a preliminary exploration, we also compared women with RAN versus BPAN.

Three hypotheses were tested. First, that the AN group will have significantly reduced GMV, white matter volume (WMV) and greater CSF volume, and that decreases in GMV will be specific to regions associated with somatosensory processing. Second, that regional brain volume differences will correlate with levels of restraint in women with AN. Third, that women with RAN compared to those with BPAN will have lower GMV in regions associated with somatosensory processing and appetitive responses and greater volume in regions associated with dietary restraint.

This article reports a novel, albeit tentative finding that there are discrete differences in brain volume between women with the subtypes of anorexia nervosa (namely restricting and binge purging subtypes) that befits the phenotypic eating behaviour of each disorder. However, due to the small numbers within the subtypes, this finding needs replication.

## Methods

### Participants

The patient group included 14 women who fulfilled DSM-IV criteria for RAN or BPAN. They were recruited from inpatient services of the South London and Maudsley NHS Trust (SLaM), between January 2005 and January 2007. Diagnosis was based on questionnaires and confirmed by a psychiatrist. The HC were 21 healthy women who had no history of an eating disorder (ED) or any other psychiatric condition and who were recruited by public advertisement. The SCID (researcher version) was used to screen for history of Axis I disorders, personality disorders and presence of an ED. The study was approved by the Joint South London and Maudsley and the Institute of Psychiatry Research Ethics Committee. All subjects gave written informed consent and were reimbursed for their participation.

### Design of the study

Participants were given two questionnaires to complete before a Magnetic Resonance Imaging (MRI) brain scan. *The Eating Disorders Examination- Questionnaire (EDE-Q *[14],*) *is a 36-item measure of dysfunctional behaviour and cognitions related to eating, with four sub-scales, eating concern, shape concern, weight concern and restrained eating, and a global eating disorder score. Questions are scored 0-6; a high score indicates greater ED pathology. This was used to confirm differences in dietary restraint between the AN subtypes and ED symptomatology between the AN and HC groups. Secondly, *the Hospital Anxiety and Depression Scale (HADS *[19] is a 14-item self-screening measure, with 7 items relating to feelings of anxiety and 7 to feelings of depression. Individual questions are scored on a 4-point scale, with higher scores indicating greater anxiety or depression. Finally, each participant was interviewed by Dr Brooks using the Structured Clinical Interview for Diagnosis (SCID).

### Structured Clinical Interview for Diagnosis-Researcher Version (SCID-R)

This was used to diagnose AN and for general screening. Duration of illness was the time between when participants reported being diagnosed with AN and the time of the scan.

### MRI data acqusition

Each participant underwent a 3D T1-weighted volumetric "inversion recovery prepared spoiled gradient recalled" (IR-SPGR) scan using a GE Signa 1.5 Tesla scanner (GE Medical Systems, Milwaukee, Wisconsin). Scan parameters were: echo time (TE) 4.9 ms; repetition time (TR) 10.8 ms; inversion time (TI) 300 ms, excitation flip angle 18°. Field of view (FOV) was 28.0 × 17.5 cm with a matrix size of 256 × 16, and 146 1.1 mm slices ('partitions') were acquired, parallel to the AC/PC line, resulting in a final voxel size of 1.1 × 1.1 × 1.1 mm.

### Voxel-based morphometry processing

Morphological changes were examined by segmentation of gray matter (GM) volumes using Voxel Based Morphometry (VBM), SPM-5 (Wellcome Dept of Cognitive Neurology, London, UK) and the BAMM package (Brain Activation and Morphological Mapping, a development of the Brain Mapping Unit, Dept of Psychiatry, University of Cambridge and the Inst. of Psychiatry, KCL, London, UK, (http://www-bmu.psychiatry.cam.ac.uk/BAMM) in native space using the unified segmentation approach [20]. After segmentation, GM probability images were "modulated" to account for the effect of spatial normalisation by multiplying each voxel value by its relative volume before and after warping. Individual GM images were normalised into Montreal Neurological Institute (MNI) standard space for analysis; to aid interpretation, reported coordinates have been converted from MNI to Talairach space using the 'mni2tal' script (http://imaging.mrc-cbu.cam.ac.uk/imaging/MniTalairach). Total CSF volumes and total GM volumes were extracted from the modulated images. Modulated images were smoothed with a 12 mm FWHM Gaussian kernel which is compatible with other VBM studies in AN [5,17]. This was done prior to statistical analysis, to reduce noise and to allow for the effects of small residual mis-registrations.

### Statistical analysis

We wished to assess the significance of differences in GMV in two separate analyses, between the AN and HC groups, and then between the RAN and BPAN. As analysis based on 3D clusters gives a more powerful measure of structural brain changes that occur over a number of contiguous voxels than statistics relying on information from a single voxel [20], we used a cluster based approach in both cases. Since no parametric distribution is known for cluster mass, a permutation based calculation is necessary to assess statistical significance in the analyses. Rather than the parametric approach available within SPM (which would have limited us to voxel level statistics), statistical analysis of brain volume contrasts between the groups was therefore implemented using BAMM, which has been used in a number of similar previous studies [21,22].

Between-group differences in GMV were estimated by fitting an analysis of covariance (ANCOVA) model at each intracerebral voxel in standard space. All covariates were chosen if they did not significantly differ between groups. In the AN vs. HC contrast the covariates were total GMV and age, whereas in the relatively homogenous AN group, to compare RAN with BPAN, the covariates were GMV, age, BMI, years of education and trait anxiety. Initially, a relatively lenient p-value (p ≤ 0.05) was set to detect voxels putatively demonstrating differences between groups and then searched for spatial clusters of such voxels and finally, tested the mass of each cluster (the sum of suprathreshold voxel statistics it comprises) for significance. Permutation based testing, implemented in the BAMM package was used to assess statistical significance at cluster level, corrected for covariates. Subsequent analysis was based on the corrected cluster level p values of 0.002 for AN v HC comparisons, and 0.01 for RAN v BPAN comparisons. These probability thresholds were set, post hoc following initial whole brain analysis at p = 0.05, according to False Discovery Rate (FDR) correction, so that reported clusters met the 'one false positive cluster' limit.

### Correlation and Regression Analyses

Simple correlation analyses were conducted using SPSS Version 18.0 (SPSS Inc. 2009, Chicago). This was done separately as a region of interest approach, in both the AN and HC groups, between those brain regions that show differential brain volume (right insula, cerebellum, bilateral parahippocampal gyrus, left fusiform gyrus, right DLPFC and left posterior cingulate), and age, BMI, lowest BMI, years of education, dietary restraint score (EDEQ), anxiety and depression scores. Brain regions were independently selected for correlation analysis using the Region of Interest extraction tool in MaRsBar via the SPM toolbox (http://www.fil.ion.ucl.ac.uk/spm/). Pearson correlations were appropriately Bonferroni corrected. Subsequently, a hierarchical regression analysis, (also done in SPSS) was performed on brain regions that showed volume differences between the AN and HC groups. This was not done between the AN subtypes as the group numbers were not sufficient. Brain volume was used as the outcome variable for each region of interest. The manually added predictor variables to the hierarchical model were restraint score (EDEQ) and current BMI, as dietary restraint may be associated with DLPFC function [23]. Following this common procedure in SPSS, the regression model was weighted for duration of illness to establish whether recovery from AN in terms of weight restoration, might be linked to GMV [7].

## Results

### Participant Characteristics

Clinical characteristics of participants are summarized in Table 1. BMI was significantly lower in the AN group (mean ± SEM: 15.57 ± 0.35 vs. 21.39 ± 0.52 kg/m2, P < 0.01). There was no significant difference in BMI between the subtypes although the self-reported lowest lifetime BMI was significantly lower in the RAN compared to the BPAN group (11.73 ± 0.38 vs. 13.36 ± 0.39 kg/m2, P < 0.01). Lowest ever BMI is usually seen as a measure of severity of illness [24]. Women with AN had a significantly lower number of years of formal education than the HC group, which might reflect periods of illness. The AN group had greater levels of dietary restraint than the HC group (3.02 ± 0.45 vs. 1.0 ± 0.22, P < 0.01), but there were no difference between the subtypes. However, although not significant, the women with BPAN did report higher restraint scores than the women with RAN, and this could reflect the subjective experience of an effort to restrain rather than actual restraint. Women with BPAN reported that they binged and purged (vomited) on a monthly basis, on average 16-22 days per month, whereas the women with RAN reported no binges or purges. Lastly, the AN group were significantly more anxious than the HC group (13.9 ± 0.83 vs. 4.38 ± 0.61, P < 0.01) and also more depressed than the HC group (10.3 ± 1.23 vs. 1.62 ± 0.39, P < 0.01), but there was no difference between the subtypes.

**Table 1:** Clinical characteristics of subjects

Variable	AN (n = 14) Mean ± SEM	HC (n = 21) Mean ± SEM	AN v. HC t-value	AN v. HC p-value	RAN (n = 8) Mean ± SEM	BPAN (n = 6) Mean ± SEM	RAN v. BPAN t-value	RAN v. BPAN p-value
**Age, years**	26 ± 1.9	26 ± 2.1	0.16	ns	26 ± 2.9	27 ± 2.6	0.9	ns
**BMI at scan, kg/m^2^**	15.6 ± 0.4	21.4 ± 0.5	8.4	<0.01	15.1 ± 0.5	16.2 ± 0.3	1.7	ns
**Lowest lifetime BMI, kg/m^2^**	12.4 ± 0.3	19.6 ± 0.3	10.6	<0.01	11.7 ± 0.4	13.4 ± 0.4	2.9	0.01
**Education (from 11 yrs old), years**	7.2 ± 0.6	11.5 ± 1.4	2.4	0.02	6.8 ± 0.6	7.8 ± 1.0	0.9	ns
**Duration Illness, years**	9.2 ± 1.9	--	--	--	9.2 ± 3.1	9.2 ± 2.2	0	ns
**EDE-Q (0-6), restrained eating**	3.0 ± 0.5	1 ± 0.2	4.9	<0.01	2 ± 0.5	4 ± 0.7	1.8	ns
EDE-Q (Q8) No. of binges month (0-6)*	1 ± 0.5	--	--	--	--	4 ± 0.8	--	--
**EDE-Q (Q22), No. of vomits in month**	5.8 ± 1.6	--	--	--	--	8 ± 2.5	--	--
**HADS- Anxiety (0-21)**	13.9 ± 0.8	4.4 ± 0.6	9.5	<0.01	13 ± 1.2	15.2 ± 1.0	1.3	ns
**HADS- Depression (0-21) **	10.3 ± 1.2	1.6 ± 0.4	7.8	<0.01	8.8 ± 1.8	12.3 ± 1.5	1.5	ns

AN = Anorexia Nervosa, HC = Healthy Control, RAN = Restricting Anorexia Nervosa, BPAN = Binge Purge Anorexia Nervosa, SEM = Standard Error Mean, BMI = Body Mass Index, EDE-Q = Eating Disorder Examination Questionnaire, HADS = Hospital Anxiety and Depression Scale, ns = non significant, *an average score of 1 on the EDE-Q, Question 8, represents binges on 1-5 days per month, whereas an average score of 4 represent binges on 16-22 days per month

### Whole Brain Tissue Volumes

There were no significant differences in total GMV, total WMV or CSF volumes between any of the groups, although GMV in the AN group was 4% lower than the HC group, and the RAN group was some 2% lower than the BPAN group. **See **Table 2.

**Table 2:** Whole brain tissue volume data in women with anorexia nervosa, restricting anorexia nervosa, binge-purge anorexia nervosa and healthy controls

Tissue	AN (n = 14) Mean ± SEM	HC (n = 21) Mean ± SEM	AN vs. HC t-value	AN vs. HC p-value	RAN (n = 8) Mean ± SEM	BPAN (n = 6) Mean ± SEM	RAN v. BPAN t-value	RAN vs. BPAN p-value
**Gray Matter Volume**, cm^3^	88.16 ± 2.55	91.80 ± 2.19	1.074	ns	87.30 ± 3.09	89.29 ± 4.58	0.374	ns
**White Matter Volume**, cm^3^	54.09 ± 1.13	52.25 ± 1.21	1.059	ns	54.43 ± 1.88	53.65 ± 1.05	0.331	ns
**CSF Volume**, cm^3^	142.25 ± 3.28	144.05 ± 2.71	0.422	ns	141.74 ± 0.44	142.94 ± 5.35	0.175	ns

AN = Anorexia Nervosa, HC = Healthy Control, RAN = Restricting Anorexia Nervosa, BPAN = Binge-Purge Anorexia Nervosa, SEM = Standard Error Mean, CSF = Cerebro-Spinal Fluid, ns = non significant

### Voxelwise Analysis of Gray Matter Differences between Groups

#### AN versus HC

**Figure 1:**
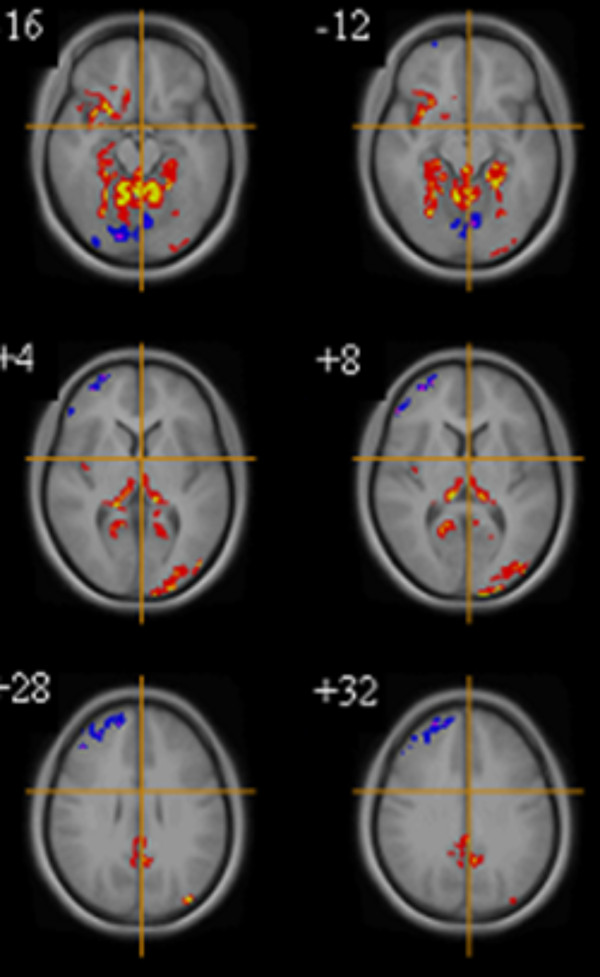
**Gray matter volume differences between all women with AN (n = 14) and healthy controls (n = 21)**. Voxel-based morphometry maps thresholded at p = 0.002 for false positives and corrected for covariates (Total gray Matter Volume, Age). Red indicates deficits in gray matter volume in women with AN compared to controls, blue indicates excesses in gray matter volume in women with AN compared to controls. Z score coordinates given are from the Talairach atlas.

After correcting for total GMV and age, the data shows that in the AN group, there were significantly lower GMVs in the left cerebellum, bilateral parahippocampal gyrus, right anterior insula, left fusiform gyrus, and right posterior cingulate (P < 0.002 for all comparisons). In contrast, a greater GMV was seen in the right dorsolateral prefrontal cortex (DLPFC) in the AN group compared to HC (P < 0.002). **See **Table 3 Figure 1.

**Table 3:** Regional gray matter volume data in women with anorexia nervosa and healthy controls

Group contrast	Brain region	BA	Laterality	p-value, corrected	Volume, cm^3^	Talairach Coordinates		
						x	y	z
						
**HC>AN**	Cerebellum	--	Left	<0.002	2.61	-3	-52	-16
	Parahippocampal Gyrus	35	Left/Right	<0.002	0.77	24	-36	4
	Anterior Insula	52	Right	<0.002	0.52	31	13	-12
	Fusiform Gyrus	18	Left	<0.002	0.52	-25	-93	4
	Posterior Cingulate	31	Right	<0.002	0.42	4	-45	32
								
**AN>HC**	DLPFC	10	Right	<0.002	0.59	34	45	28

HC = Healthy Control, AN = Anorexia Nervosa, BA = Brodmann Area, DLPFC = Dorsolateral Prefrontal Cortex.

#### RAN versus BPAN

**Figure 2:**
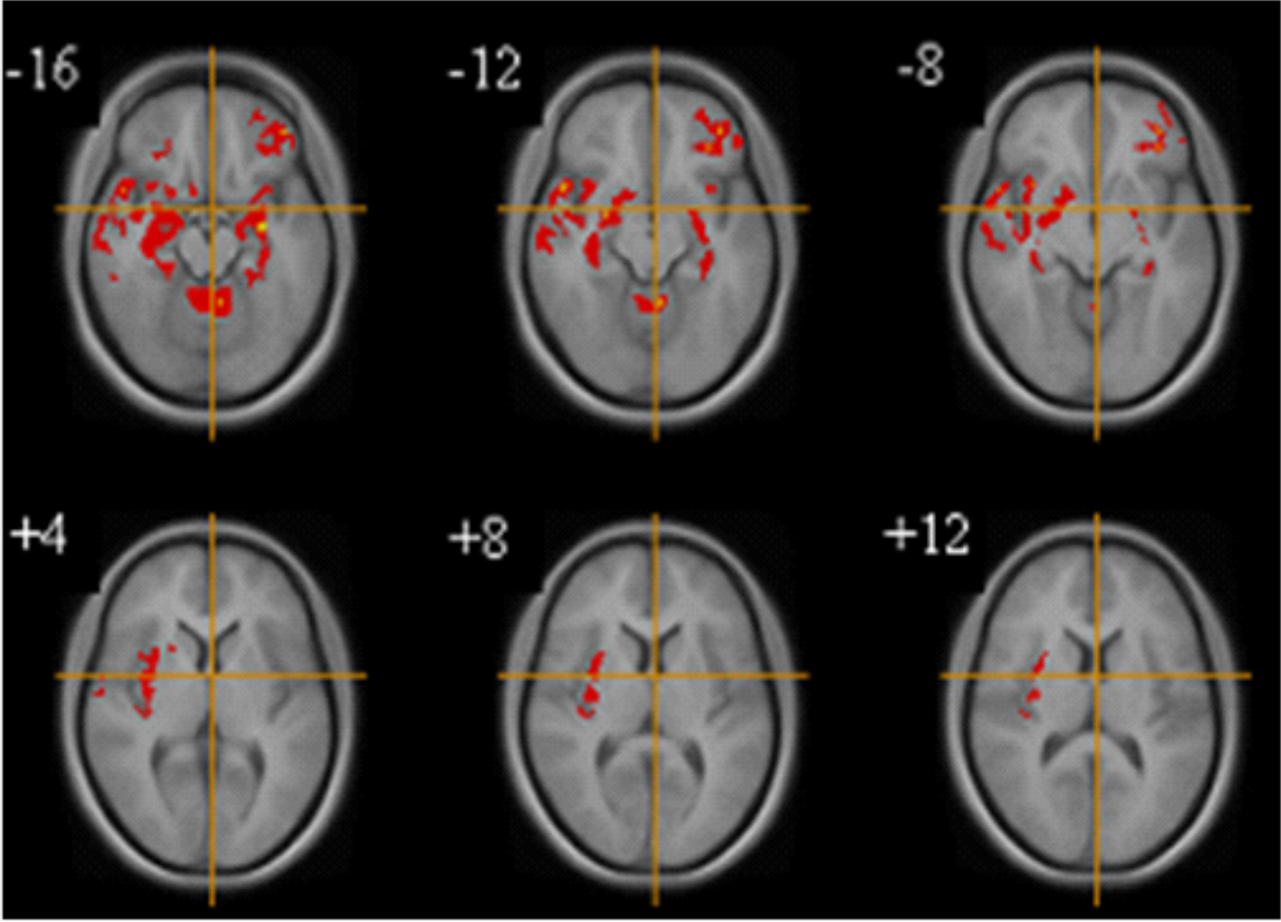
**Gray matter differences between women with restricting (n = 8) and binge purge AN (n = 6)**. Voxel-based morphometry maps thresholded at p = 0.01 for false positives and corrected for covariates (Total gray matter volume, Age, BMI, Years Education, Trait Anxiety). Red indicates deficits in gray matter volume in women with RAN compared to BPAN. Z score coordinates given are from the Talairach atlas.

After correcting for total GMV, age, BMI, years of education and trait anxiety, the data shows that relative to the BPAN group, the RAN group, had significantly lower GMV in the left cerebellum, bilateral parahippocampal gyrus, right anterior insula and left orbitofrontal cortex (OFC) (P < 0.001). **See **Table 4 Figure 2.

**Table 4:** Regional gray matter volumes in subtypes of anorexia nervosa (restricting and binge purging)

Group contrast	Brain region	BA	Laterality	p-value, corrected	Volume, cm^3^	Talairach Coordinates		
						**x**	**y**	**z**
						
**RAN > BPAN**	--	--	--	--	--	--	--	--
**BPAN> RAN**	Cerebellum	--	Left	<0.01	0.77	-9	-54	-16
	Parahippocampal Gyrus	35	Left/Right	<0.01	0.52	-24	-10	-12
	Anterior Insula	13	Right	<0.01	0.21	40	-3	4
	OFC	11	Left	<0.01	0.19	-35	41	-8

RAN = Restricting Anorexia Nervosa, BPAN = Binge-Purge Anorexia Nervosa, OFC = Orbitofrontal Cortex, BA = Brodmann Area.

#### Correlation and Regression Analysis

**Figure 3:**
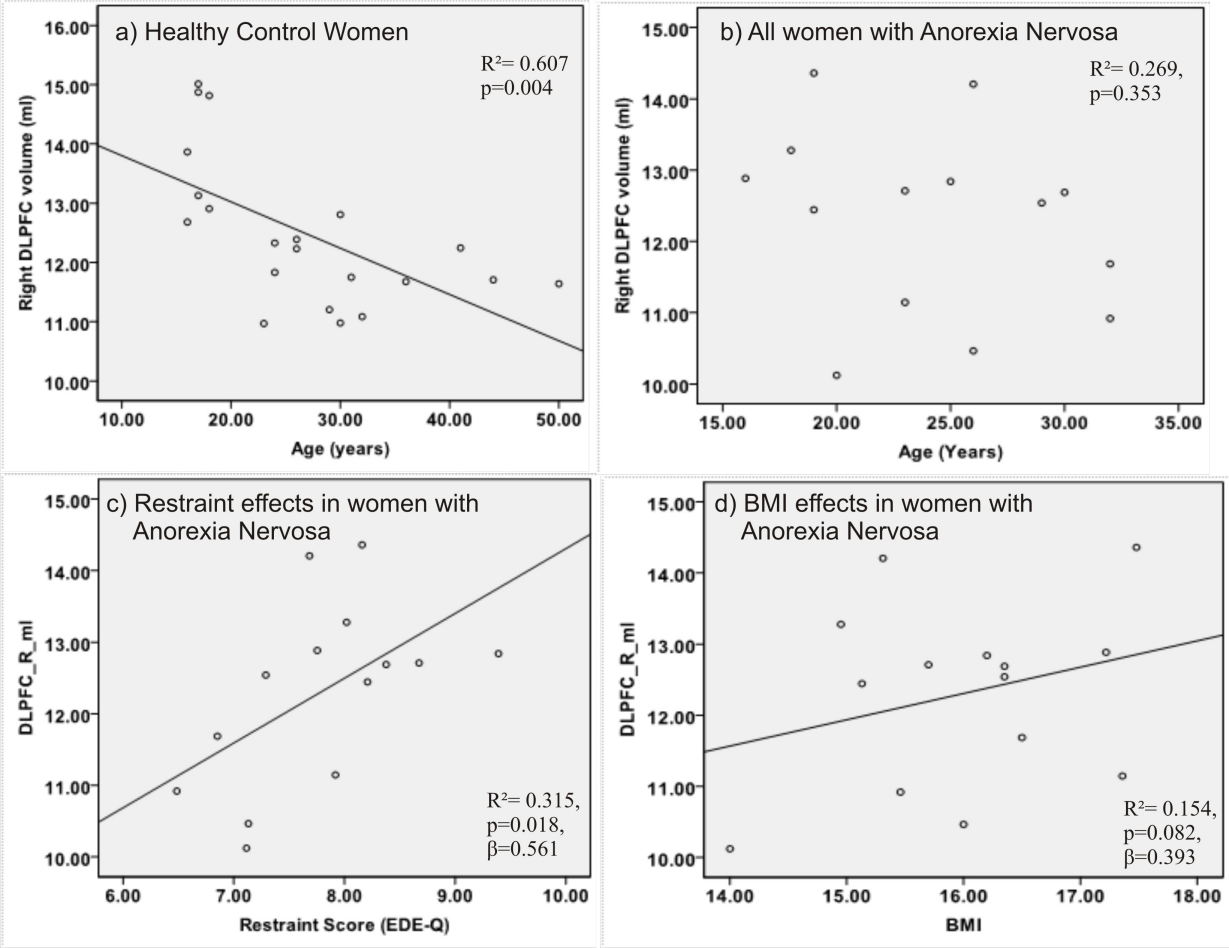
**Regression analyses with brain volumes**. Negative correlation with age and right DLPFC volume in **a**) healthy women; **b**) no correlation in women with AN; **c**) regression analysis: first level predictor restraint (EDE-Q); **d**) second level predictor current BMI (log transformed) of right DLPFC volume in women with AN; both predictors were weighted for duration of illness.

There was a significant negative correlation between age (which did not differ between the groups) and GMV in the right DLPFC in the HC (R^2^= 0.607, p = 0.004, Figure 3), but not in those with AN (R^2^= 0.269, p = 0.353, Figure 3). In no other brain areas were volumes correlated (after Bonferroni correction) with age, BMI, lowest BMI, years of education, dietary restraint, anxiety and depression. A hierarchical regression analysis was carried out in an attempt to explain which variables in the AN group contributed to the larger right DLPFC volume, that was identified in the contrast with HC women. As longitudinal VBM studies have reported that recovery from AN can impact on brain structure [7], the regression analysis was weighted against duration of illness. As it has been proposed that excessive DLPFC activation underlies the excessive dietary restraint seen in women with AN [23] the dietary restraint score was inserted as the first level predictor. The level of dietary restraint, weighted for duration of illness, significantly explained 32% of the variance in right DLPFC volume in the AN group (R^2^ = 0.315, p = 0.018, β = 0.561, Figure 3). As dietary restraint is a mechanism suggested to control appetitive processes [25], current BMI was used as a second level predictor. On its own and weighted for duration of illness, current BMI explained 15% of the variance in the right DLPFC volume in the AN group and this almost reached significance (R^2^ = 0.154, p = 0.082, β = -0.393, Figure 3). When combined, current BMI and dietary restraint significantly explained 57% of the variance in the right DLPFC volume (R^2^ = 0.566, p = 0.008, β (BMI) = 0.477, β (restraint) = 0.584). **See **Table 5.

**Table 5:** Regression analysis values for outcome variable: right dorsolateral prefrontal cortex (DLPFC) volume (weighted for duration of illness)

**Model**	**Independent variable(s)** **(Weighted for duration of illness)**	**R^2^**	**p-value**	**β**	**95% Confidence Intervals for β**	
					**Upper Bound **	**Lower Bound**
					
**1**	Restraint_EDEQ	0.315	0.018	0.561	1.547	-1.335
**2**	BMI (log)	0.154	0.082	0.393	0.62	-0.325
**3**	Restraint BMI (log)	0.566	0.008 Ns	0.653 0.269	1.56 0.95	0.082 0.023

R^2^ = Coefficient, EDEQ = Eating Disorders Examination Questionnaire, BMI = Body Mass Index.

## Discussion

This study examined total and regional brain volumes in women currently ill with AN as well as in the subtypes. We found lower regional GMV in the right anterior insular cortex, bilateral parahippocampal gyrus, left fusiform gyrus, left cerebellum and right posterior cingulate and greater GMV in the right DLPFC. Right DLPFC volume was linked to age-related reduction in HC but not AN, and restraint scores predicted right DLPFC volume in AN. Thus, core pathology in AN (cognitive restraint of appetitive processes) might conserve normal age-related atrophy effects in the DLPFC, and may underlie other cognitive traits in AN, such as inflexible thinking and obsessions about shape, weight and eating. Regional differences in GMV were also found between the subtypes: those with RAN (compared to BPAN) had reduced GMV in the right anterior insular cortex, bilateral parahippocampal gyrus, left cerebellum and left OFC, that is, regional differences in GMV substantially mirrored those seen between the AN and HC groups. We did find small GMV reductions between the AN group and healthy controls, but these findings, including differences in WMV and CSF were not significant. These data were surprising, given that previous studies consistently report global reductions, e.g. [3-6]. Thus, our results were probably confounded by our small group numbers, particularly in the subgroup contrasts, and makes replication with larger subgroup numbers necessary. Nevertheless, our preliminary findings, especially regarding brain volume differences between the subtypes of AN, provide regions of interest for future studies.

In the AN group there was a lower GMV in the right anterior insular cortex, which was also seen in the RAN vs. BPAN comparison. Such reductions have previously been reported in women with AN [5] and in the operculum, an area adjacent to the insular cortex [17] and furthermore, some gray matter deficit remains following recovery [16]. We also observed a reduction in GMV in the OFC (an area associated with 'sensory-specific satiety' [26]): this is an area which has been reported to show increased GMV in people with bulimia nervosa and binge eating disorder [27]. From these observations, it can be proposed that reduced insular GMV may alter somatosensory processing and this may be more pronounced in RAN than BPAN. This is in line with other studies also showing regional reductions in somatosensory regions (e.g. temporo-parietal), that may also be related to core psychopathic traits, such as deficits in attention to detail and drive for thinness [5,17], and may contribute to homeostatic and body image problems [6,10]. Secondly, in combination with the reduction in the OFC, reduced volume in the insular cortex (or in the operculum, an area adjacent to the insular cortex), may alter the ability of people with AN to feel hunger and to appreciate the 'appetitiveness' of food.

In the AN group, reduced left fusiform gyrus and right posterior cingulate GMV were seen. The fusiform gyrus is associated with recognition of familiar objects [28] and the posterior cingulate with spatial awareness and expectancy [29] i.e. they may be part of a network involved in accurate object recognition. Women with AN display a cognitive bias for food images [30], and this may be associated with changes in these regions. From our data and other findings relating to reductions in the ACC [17], it can be hypothesised that in women with AN, cognitive bias for food stimuli could in part be driven by an imbalance that favours PFC-related attentional mechanisms over fusiform-driven spatial recognition systems, particularly for appetitive stimuli.

Reduced cerebellar GMV was seen in the total AN group, as well as a reduced GMV in the left cerebellum in the RAN group compared to the BPAN group. This is in line with a recent study finding reduced cerebellar volume that was most pronounced with longer duration of illness [10]. Given that our AN patients had an average illness duration of 9 years, it seems that the cerebellum is a key region for starvation-related atrophy. Brain imaging studies report reduced activation to food images in the cerebellum of patients with AN [31]. Given that the cerebellum has a prominent role in feeding behaviour [32] and animal studies have shown that lesioning alters feeding behaviour and results in weight loss [33,34], these data suggest that reductions in cerebellar volume (in AN and also in the RAN vs. BPAN groups) contribute to the individuals' ability to maintain a low weight, and thus to the maintenance of illness.

Reduced bilateral parahippocampal GMV in AN has been reported [11] and in the present study, the reductions were greater in the RAN compared to the BPAN group. The hippocampus is involved in learning and memory [35], and, for example, memory for specific attributes of recently eaten foods [36]. The hippocampi are also involved in keeping in mind previously determined goals, and thus may combine with working memory function associated with the DLPFC [37]. The observed structural changes might alter hippocampal-DLPFC interactions although one report of decreased hippocampal volume found no deficits in overall memory performance [8]. Increased DLPFC activation in response to food images in women with AN has been reported [31] and data have also shown that healthy women (relative to men) have greater dietary restraint and greater activation of the DLPFC in response to food images [38]. Thus, increased reliance on DLPFC-associated working memory (instead of hippocampal-related spatial memory) when dealing with food, may contribute to the rigid and strategic cognitive style associated with AN [39].

That increased right DLPFC volume in the total AN group might be associated with excessive dietary restraint is supported by the regression analysis which shows that BMI at the time of scan and dietary restraint score together predict 57% of the variance in right DLPFC GMV. The regression analysis also shows that in the AN group, increased BMI at the time of scan is associated with higher dietary restraint, possibly because they feel more necessity to restrain intake as BMI increases. Furthermore, we chose to weight our regression analysis for duration of illness, because there is minor evidence that core psychological disturbances in AN, such as excessive cognitive restraint of appetite (linked to DLPFC activation) become more embedded as the disorder progresses, e.g. [40]. Additionally, in the HC group, there was a significant decrease in right DLPFC GMV with age. This is in accord with other studies [41,42] and, perhaps rather surprisingly, suggests that the difference between the AN and HC groups is due to the maintenance of GMV in the women with AN, that is, the long term use of cognitive strategies to inhibit appetite may be associated with increased neural growth. This is in accord with findings that employment of cognitive strategies during a course of CBT may be linked to reversal of GMV atrophy in a psychiatric population e.g. a group with chronic fatigue [43].

Functional brain imaging studies demonstrate that women with eating disorders have greater activation when thinking about eating food in the right DLPFC [44] and activation in this region is also increased in response to food in women with AN who have regained a normal weight [45]. Furthermore, stimulating the DLPFC using repetitive Transcranial Magnetic Stimulation (rTMS) increases restraint in those who are prone to binge eat [46]. Moreover, stimulating the DLPFC with TMS improves working memory performance [47], and there is some preliminary evidence that women with AN perform better on a working memory task [48,49]. Thus, the DLPFC may play a key role in pathological working memory-related cognitive restraint of appetite in those with eating disorders. As we have shown, the DLPFC seems prone to normal age-related atrophy, but in women with AN, GMV in this region is preserved, possibly with overuse of cognitive strategies to restrain appetite (e.g. working ruminations about eating, concerns about shape and weight, and drive for thinness). Future studies should explore the relationship between DLPFC structure and function, working memory and restraint of appetite in those with AN.

It is unclear whether observed differences are a cause or a consequence of illness. This is important because of prolonged weight loss/poor nutrition and should also be borne in mind when comparing the RAN versus BPAN groups, as the level of malnutrition/severity of illness may be greater in the RAN group. That the differences are regional may simply reflect regional differences in vulnerability to prolonged energy deficits rather than changes associated with the psychopathology. It is of note however, that we have observed an increase in GMV in the DLPFC. This regional increase could be a trait effect associated with the risk of illness but it might also have arisen from prolonged use of cognitive techniques to control appetite as this has been reported to influence GMV [41]. In general, if the regional differences in GMV are traits, they may be risk factors for AN and its subtypes and if they are state effects, they may contribute to the maintenance of illness.

Additionally, one must take in to account the differences in clinical syndromes when comparing the subtypes of AN, in that people with RAN are able to restrict their food intake, whereas those with BPAN engage in sporadic episodes of over-eating. Differences in food intake between the subtypes may potentially influence the severity of emaciation effects on the brain. Furthermore, due to the commonly observed 'cross-over' in the 'current episode' between the subtypes, it is suggested that for the DSM-V the subtype behaviour should be present for the last three months [50]. To ensure that we included both those with clear RAN and BPAN diagnoses, we included those that had the subtype diagnosis for at least three months. However, in both subtypes of AN, sufferers exhibit severe cognitive disturbances, such as excessive perfectionism, asceticism [51], cognitive rigidity and deficits in set-shifting, which also pertains to attention to detail [52], and ruminations, obsessions about food and excessive concerns about weight and shape [53]. Perhaps the most prominent factor differentiating the subtypes, therefore, is in the level of cognitive restraint exerted over appetitive responses. And although the data is preliminary and exploratory at this stage, we suggest that cognitive rumination associated with working memory performance and DLPFC activation, conserves normal age-related atrophy in the DLPFC. Thus, excessive dietary restraint, associated with activation of the DLPFC, is potentially a biomarker for disorder severity.

There are also reports of functional neuroanatomical differences between the subtypes of AN in the prefrontal cortex and in somatosensory regions such as the insula, for example, reduced somatosensory but greater prefrontal activation in RAN [45]. However, despite evidence from fMRI studies that brain activation correlates with global brain volume [13] it is not known whether functional differences are associated structural anatomical differences in women with AN. Therefore, it is important for future functional brain imaging studies of AN to correlate Blood Oxygen Level Dependency (BOLD) signal with gray matter volume.

There are four other VBM studies that most closely resemble ours [5,6,10,17], in that they demonstrate, using similar statistical techniques (e.g. optimized high resolution T1-weighted MRI, global and regional analysis), reduced brain volumes in the cerebellum, frontal and temporal regions. One study also compared the same AN group during illness and after recovery, and found increases in global GMV and reductions in temporal and supplementary motor regions after recovery [5]. In contrast to our study, some of these VBM studies have examined larger groups of anorexia nervosa patients, (ranging from 12 to 29), and some have focused on adolescents at the early stages of the illness [5,6]. Additionally, unlike in this study, correlations have been shown with short duration of illness, BMI and hypothalamus reduction, and a long duration of illness with cerebellar reduction [10], and longer copy time on the Rey Complex Figures Task with reduced global GMV [5]. Furthermore, 'drive for thinness' is linked by one VBM study to brain volume in the right inferior parietal lobe [17]. Moreover, all these studies found global GM reductions in females with AN [5,6,10,17], and although not significant (likely due to small numbers) we did find reductions also. Thus, many psychopathological traits in AN correlate with changes in brain volume, in regions we also describe. However, none of these previous VBM studies have examined the relationship between severe dietary restraint and regional brain volume in relation to the different subtypes of AN, which is the uniqueness of the present study.

There are some limitations to this preliminary study, for example, the data are based on a relatively small sample and despite stringent voxel thresholds and co-variate correction, caution is necessary when generalizing to larger populations. Five HC women were older than the oldest woman with AN, which strengthened the correlation, although the correlation was still significant after removing them, and the average age between groups was not significantly different. We chose not to include lowest lifetime BMI in our calculations because, due to the average duration of illness (9 years), and given that eating disorder diagnoses often fluctuate between AN and BN [54], we decided that BMI at time of scan would be a more accurate indicator of current effects of emaciation on brain volume. Nonetheless, we acknowledge that some atrophy effects could be attributed to the lowest lifetime BMI, as has been shown elsewhere, e.g. [7,16] and we suggest that this should be explored further in future studies that compare the subtypes of AN. The use of VBM limits our interpretations of possible neuroplasticity effects, and more longitudinal and functional studies are required. Lastly, the VBM technique is designed to study localised differences and other techniques may be more appropriate for detecting global ones.

## Conclusions

This study examined brain volumes in women with AN and its subtypes. Regional differences in GMV were identified in areas linked to appetitive, somatosensory and memory processing associated with AN and which differentiate RAN from BPAN. Age-related decreases in the right DLPFC were not seen in women with AN, and this may be attributed to the employment of cognitive restraint of appetite over their illness duration. Conversely, since this is correlational and not causal it could also mean that a larger brain volume in this region leads to the development of cognitive restraint strategies. Whether these differences are state or trait related is unclear but in either case, they may contribute to the maintenance of illness.

## Competing interests

The authors have no competing interests to disclose.

## Authors' contributions

SJB, ICC, and JT designed the study. SJB and GB analyzed the data. SJB and JT recruited patients. SJB, GB, OGOD, MB, SCRW, CB, HBS, JT and ICC contributed to writing the paper. SJB collected data and conducted the experiments. All authors had access to all data in the study and take responsibility for the integrity and accuracy of the data analysis. All authors have read, and approve the manuscript.

## Pre-publication history

The pre-publication history for this paper can be accessed here:

http://www.biomedcentral.com/1471-244X/11/179/prepub
